# Unmanned Aerial System Integrated Sensor for Remote Gamma and Neutron Monitoring

**DOI:** 10.3390/s20195529

**Published:** 2020-09-27

**Authors:** Alexander Barzilov, Monia Kazemeini

**Affiliations:** Department of Mechanical Engineering, University of Nevada Las Vegas, Las Vegas, NV 89557, USA; kazemei2@unlv.nevada.edu

**Keywords:** remote sensing, UAS, plug and fly CLYC sensor, neutrons, gamma spectroscopy

## Abstract

Tools for remote radiation sensing are essential for environmental safety and nuclear power applications. The use of unmanned aerial systems (UASs) equipped with sensors allows for substantially reducing the radiation exposure of personnel. An ambient temperature Cs_2_LiYCl_6_:Ce^3+^ (CLYC) elpasolite scintillation sensor for simultaneous gamma and neutron measurements was designed as a user-friendly “plug and fly” module integrated into an octocopter robotic platform. Robot Operating System (ROS) was used to analyze the sensor’s data. The measured CLYC’s energy resolution was <5% at 662 keV gamma rays; neutron flux was measured using ^6^Li(*n*,α)*t* reaction. Time and GPS data were combined with radiation data in the ROS, supporting real time monitoring and assessment tasks, as well as radiation source search missions. Because UASs can be irradiated, radiation damage of the sensor and robot’s electronics was estimated using FLUKA code.

## 1. Introduction

Radioactive materials can be released into the environment because of natural disasters or accidents at nuclear installations, or a combination of both. For example, an earthquake and tsunami led to a disaster at the Fukushima Daiichi Nuclear Power Plant in Japan [1,2,3,4]. This accident caused long-term radiological pollution of the power plant location and its adjacent areas, as well as the ocean water, requiring costly remediation efforts. Displaced or lost industrial and medical sources of ionizing radiation is another pathway of environmental contamination [5,6,7,8,9]. These man-made hazards cause irradiation to personnel, the public, and the environment. Radiation monitoring is essential for environmental safety and radiation safety tasks in industry and nuclear power generation. The use of mobile robotic platforms equipped with radiation sensing and navigation tools enables remote monitoring of hard-to-reach hazardous zones and wide areas, decreasing risks of radiation exposure of personnel.

Unmanned aerial systems (UASs) are flying robotic platforms that can incorporate sensors for radiation monitoring in the field conditions [10,11]. The use of UASs equipped with navigation modules allows dynamic tracing of the radiation flux and contamination analysis in space and time, e.g., dose mapping in a wide area [12,13,14]. The deployment of several UAS platforms simultaneously enables cooperative sensing technologies, such as “contour mapping” of complex boundaries of a contaminated zone, and the localization and quantification of radiation sources.

To make it possible, radiation sensors must be integrated with a robotic platform, taking into account the available power, data processing, and communication capabilities. To deploy UASs equipped with sensors in field conditions, the user-friendly “plug and fly” option of hot attachment and unplugging of the sensors into a robot is desirable [15,16]. Furthermore, to decrease the size of data packages transmitted from the UAS to a ground station or to other robots, analysis of the sensor’s signals must be done onboard [17,18,19]. In remote measurements of radiation, gamma rays and neutrons are relevant (charged particles such as electrons and alpha particles are attenuated by thin layers of shielding and by air at short ranges). Usually, separate sensors are utilized for detection of gamma rays and neutrons, some of them with a cryogenic cooling, thus requiring bulky modules and setting size, weight, and power limits for deployment as a part of robotic systems. Dual-radiation detectors are being studied for neutron-photon imaging systems [20,21]. Simultaneous detection of gamma rays and neutrons by a single ambient-temperature sensor is necessary for the UAS-based sensor package.

To address this need, a Cs_2_LiYCl_6_:Ce^3+^ (CLYC) elpasolite sensor was designed for simultaneous neutron detection and gamma spectroscopy and integrated into a UAS platform. The sensor can operate in ambient temperatures without cooling, which is crucial for field deployment. Robotic platforms can be subjected to high levels of radiation in contaminated zones. Because of the radiation damage, the UAS’s operational time is affected. Irradiation causes dislocation loops, voids, and macroscopic defects in the electronics components of the UAS. The robotic platform should complete as many tasks as possible under irradiation. The evaluation of radiation damage in electronics and sensor materials is important: the shielding can be added to the most vulnerable components of the robot, and its operations can be planned according to the dose conditions.

This research was presented (in part) at the 6th International Electronic Conference on Sensors and Applications (ECSA-6, 15–30 November 2019) and published in MDPI Proceedings [22]. This paper is an expanded and extended version of the proceeding’s publication.

## 2. Materials and Methods

### 2.1. Mobile Unmanned Aerial System

**Figure 1:**
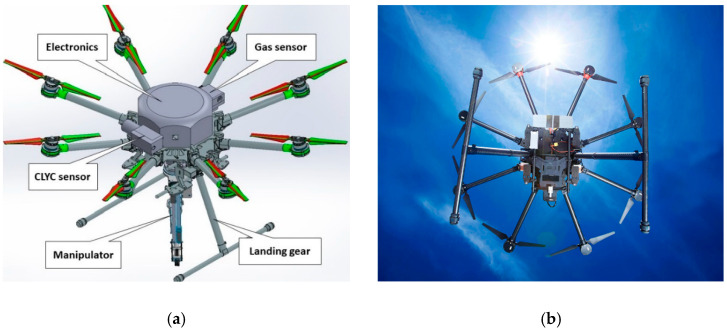
(**a**) Rendering of the unmanned aerial system (UAS) with attached payload; (**b**) aerial robotic platform in flight.

The DJI-S1000 kit (DJI, Shenzhen, China) [23] was utilized to design the UAS platform (see Figure 1). The diagonal wheelbase of the 4.4-kg octocopter frame is 104.5 cm. The frame has eight 38.6-cm-long arms with the 38-cm-diameter propellers and electric motors (the 4114 Pro model). The UAS’s landing gear can be raised in flight. The UAS equipped with a 6S lithium battery can fly 15 min with a 6.8 kg payload. The UAS can carry external attachments—several sensors (radiation and gas sensors, a thermal imaging camera) and devices such as a manipulator and sampling gear.

The UAS is remotely controlled by an operator using a graphical user interface (GUI) or a radio controller (RC), and by a ground station’s computer that enables automatic implementation of the planned tasks. The ground station computer generates commands that are transmitted to a UAS’s onboard Linux minicomputer (the Odroid-XU4 model) running Robot Operating System (ROS) [24]. ROS is a software framework designed for robotic applications. It includes libraries and tools consisting of a few communicating nodes using a publishing or subscribing messaging model.

The minicomputer is the robot’s central data hub. It controls the CLYC neutron/gamma sensor, a gas sensor (Smart Sensor Tech, Bowling Green, KY, USA), an (optional) lidar (Hokuyo Automatic, Osaka, Japan), and a 915 MHz radio transceiver (Helixiongdi Technology, Shenzhen, China). It is also connected to the flight controller Pixhawk 2.1 [25] that includes the isolated and dampened components—a flight management unit and an inertial measurement unit with a built-in heating system and robust connectors that resist shocks and diminish noise. Pixhawk executes the low-level flight control. Reference velocity data can be provided by the minicomputer. The position-feedback data can be supplied by GPS (if available) or PX4 Flow, a combined optic flow and sonar sensor, if a GPS signal is unavailable. The Lidar supports obstacle avoidance in the autonomous mode.

The radiation data streamed by the CLYC sensor was time-stamped and supplied with the UAS’s position data, using the real time kinematic (RTK) GPS positioning technology. The RTK GPS is a navigation technique that enhances the precision of position data derived from satellite-based positioning systems. It is based on measurements of the phase of the signal’s carrier wave, using a single reference station to provide the real time corrections [26,27].

**Figure 2:**
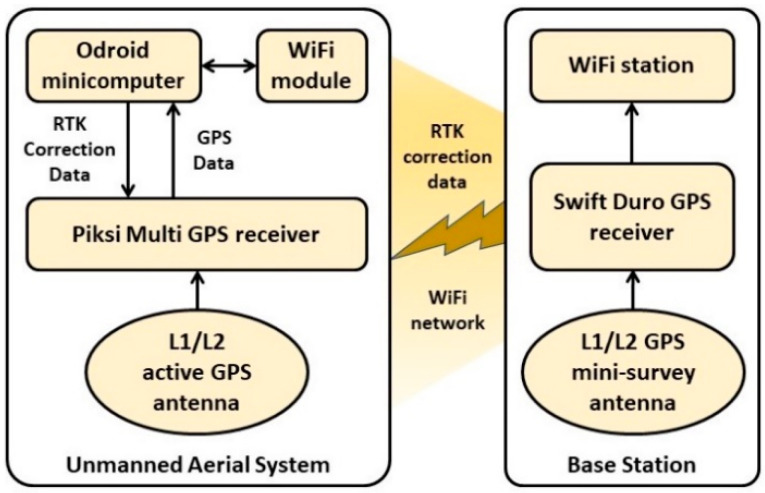
Real time kinematic (RTK) GPS operation diagram.

The base station is equipped with a Swift Duro GPS receiver (Swift Navigation, San Francisco, CA, USA) [28]. The base station has precisely surveyed coordinates. The UAS platform is equipped with a Swift Navigation’s Piksi Multi GPS receiver [29] and L1L2 antenna (Maxtena, Rockville, MD, USA) [30]. Correction data for ionosphere error calculations was communicated to the GPS receiver of the UAS platform from the base station. It should be noted that several UASs (a swarm) can utilize a single RTK GPS base station for cooperative flight missions. The RTK GPS system supports raw data measurement rates up to 20 Hz, and RTK position output up to 10 Hz. The operation diagram of RTK GPS is shown in Figure 2.

### 2.2. Ambient Temperature Gamma/Neutron Sensor

The gamma/neutron radiation sensor is based on an elpasolite CLYC scintillator (3.31 g/cm^3^ density), with bright output that allows for simultaneous neutron and gamma ray measurements using a single sensor. Subject to the lithium isotopic composition of the crystal, the CLYC sensor can be used for thermal neutron measurements via ^6^Li(*n*,α)*t* reaction or for fast neutron measurements using ^35^Cl(*n*,*p*)^35^S reaction with *Q* value of 615 keV, or for both. The thermal neutron’s peak is at the 3-MeV gamma equivalent energy (GEE) in the spectrum. In the (*n*,*p*) reaction case, the emitted proton’s energy is 615 keV plus the incident neutron’s energy. Hence, the neutron energy can be determined based on the measured full-energy gamma peak value.

The CLYC scintillator allows gamma ray spectroscopic measurements as other materials of the elpasolite family [31,32,33]. The CLYC scintillation yields for gamma rays and neutrons are as follows: 2 × 10^4^ photons for a 1-MeV absorbed gamma ray and 7 × 10^4^ photons for an absorbed neutron. The CLYC’s scintillation-light wavelength curve extends from 275 nm to 450 nm; the peak is at 370 nm. The CLYC’s refractive index is 1.8 at 405 nm.

The scintillation process in a CLYC crystal has three distinct decay components: (1) the core-to-valence luminescence (CVL) with the wavelength range from 250 nm to 350 nm and 2 ns decay time constant; (2) prompt Ce^3+^ emission with 350–450 nm range and 50 ns decay constant; and (3) cerium self-trapped excitation (Ce-STE) with 1000 ns decay constant. Neutrons induce the scintillation emission in the CLYC that de-excites via the Ce-STE decay. The first two components are due to gamma-induced processes in the CLYC. Significant differences in decay times in the CLYC scintillator makes it possible to discriminate neutron and gamma events using pulse shape discrimination (PSD) methods [34,35].

**Figure 3:**
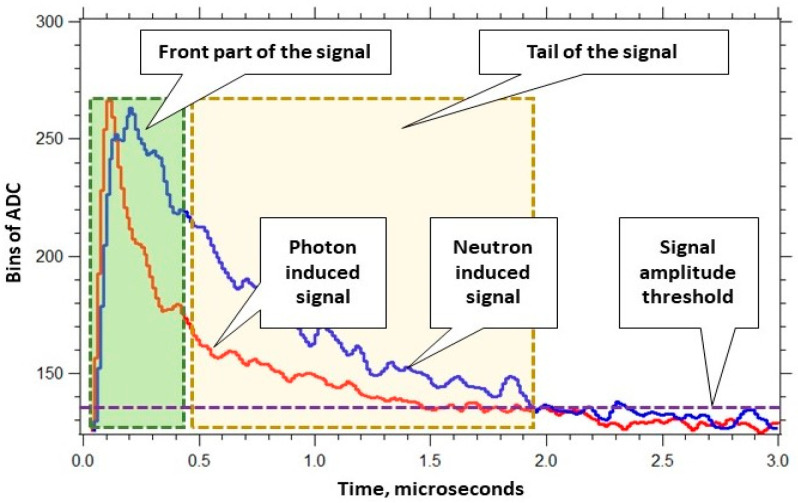
Cs_2_LiYCl_6_:Ce^3+^ (CLYC) sensor: recorded neutron and gamma ray waveforms.

Waveforms of digitized neutron-induced and gamma-induced signals in the CLYC are displayed in Figure 2. The waveforms were processed to generate three values recorded in a list mode: (1) a signal’s start time; (2) an integral calculated under the whole waveform (that is proportional to the energy of absorbed radiation); and (3) an integral calculated under the front part of the waveform. These three values of the list mode data were used for the neutron/gamma PSD that works as follows. First, the radiation identification value (ID) was determined for each waveform as a ratio of the integrals under its tail part and front part (see Figure 3). Neutron waveforms are longer tailed than the gamma waveforms, thus generating greater ID values. Second, the radiation IDs were used to segregate waveforms into two groups: neutron events and gamma events.

**Figure 4:**
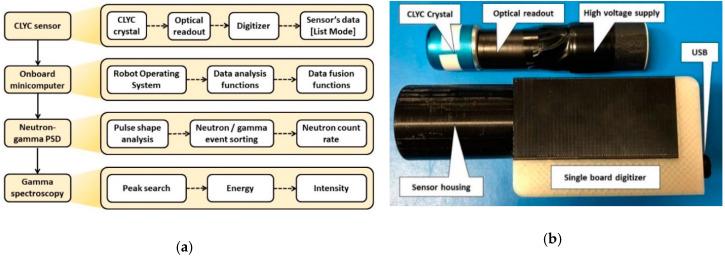
(**a**) Scheme of CLYC sensor operation; (**b**) sensor package.

The sensor package was designed utilizing a 2.54-cm-diameter, 2.54-cm-long cylindrical CLYC crystal. The CLYC crystal (RMD, Watertown, MA, USA) was housed in 3-mm-thick aluminum enclosure with a quartz window on its base. The crystal was coupled to a super bialkali photomultiplier tube (Hamamatsu, Naka-ku, Japan) rated for CLYC’s scintillation wavelengths, and a miniature high voltage generator with eMorpho digitizer [36] (Bridgeport Instruments, Austin, TX, USA) connected through the USB cable to the minicomputer was used to process the sensor’s signals. The components of the sensor were packaged in a plastic housing designed to be easily attached to the UAS. The CLYC sensor’s operation scheme and the sensor package are shown in Figure 4a,b.

### 2.3. Sensor Integration with the UAS

**Figure 5:**
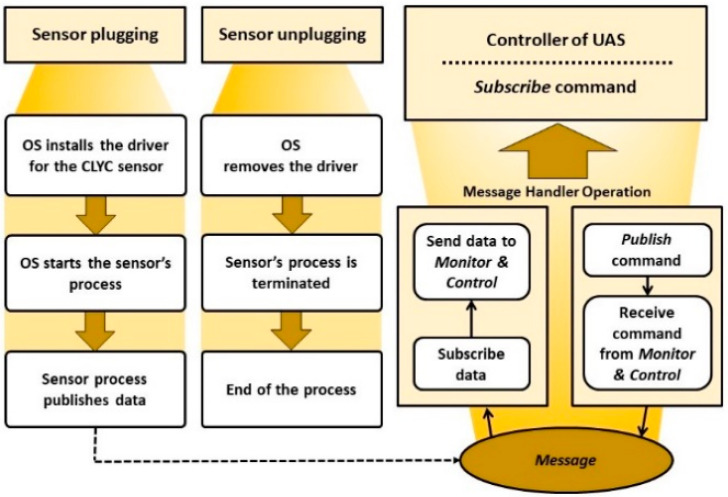
Scheme of “plug and fly” operation of the CLYC sensor.

**Figure 6:**
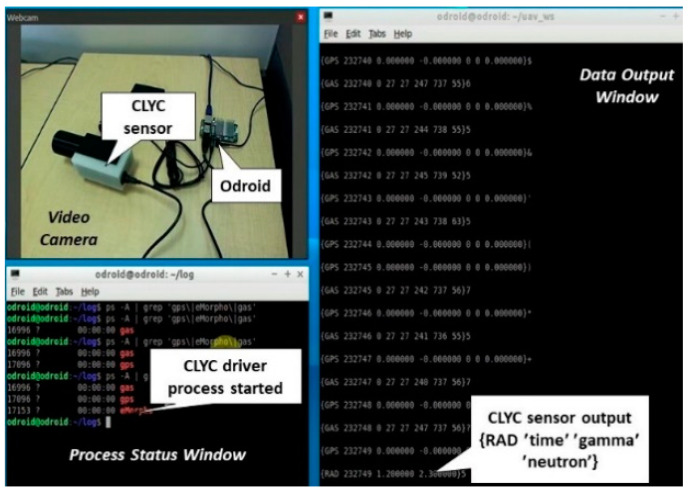
Laboratory testing of driver processes and the CLYC sensor’s data stream.

The “plug and fly” approach was used in the design of the CLYC sensor integration with the UAS platform. It provides “hot plugging” and “hot unplugging” of the sensor via the USB interface of the UAS. When the sensor is plugged in and powered on, the operating system (OS) identifies its type (several different sensors can be used at the same time). Then, the OS installs a sensor’s driver, starts the measurement, starts processing and analyzing the measured data, and then publishes them. The scheme of “plug and fly” sensor operation in the ROS environment is shown in Figure 5. The integrated CLYC sensor was tested in the “plug and fly” mode using ROS. Figure 6 illustrates the driver processes and the sensor’s data stream, including the time stamp and RTK GPS data fusion with the radiation readings.

### 2.4. Radiation Damage Modeling

The UAS can be exposed to high levels of radiation that can cause damage to vulnerable parts (such as electronic components) and even terminate the platform’s operation. To optimize the UAS’s operational lifetime, it is important to estimate the radiation damage of its electronic parts. Displacement per atom (DPA) represents the displacement damage in a material as a result of the deposited ionizing radiation. This would affect the macroscopic crystal defects. DPA embodies the damage-based exposure unit, which is the number of times the atoms have been displaced from their original lattice sites [37].

**Figure 7:**
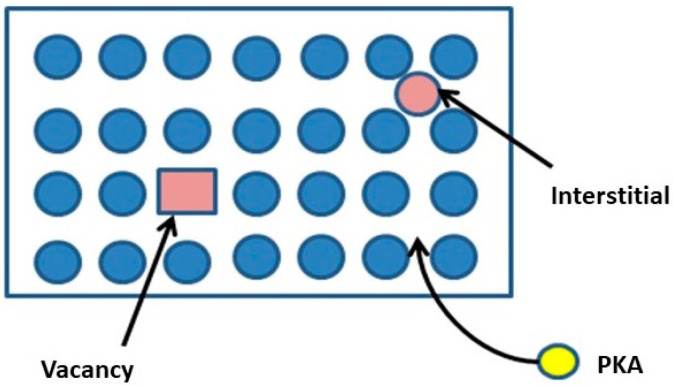
Frenkel pairs.

The DPA is directly linked to the number of Frenkel pairs. The displacement occurs by a primary knock on atom (PKA) caused by the elastic scattering of incident particles. Frenkel pairs are crystallographic defects when an atom leaves the original lattice site, creating a vacancy, and then lodging at a different location, becoming an interstitial. Therefore, the Frenkel pairs are called vacancy-interstitial pairs (Figure 7). The DPA is calculated as *DPA* = (*A*/*N*_A_ *ρ*) *N*_F_, where *ρ* is the density in g/cm^3^, *N*_F_ is the number of Frenkel pairs, *N*_A_ is Avogadro’s number, and *A* is the mass number.

The FLUKA code [38,39,40] was used to calculate the DPA in various materials of the robot’s electronics parts using the Kinchin–Pease damage model [41] modified by Norgett, Robinson, and Torrens [42]. Lattice defects are formed when the gamma rays and neutrons interact with the crystalline materials. To safeguard the robotic platforms from the radiation damage, three options are (1) to minimize time at the contamination area; (2) to keep distance from the source of contamination, or (3) to use a radiation shielding of the electronic parts. While the time and distance parameters can be planned for the robot performing the tasks in a contamination zone, radiation shielding of the electronic components was modeled using FLUKA.

## 3. Results and Discussion

### 3.1. UAS Positioning Accuracy Measurement

**Figure 8:**
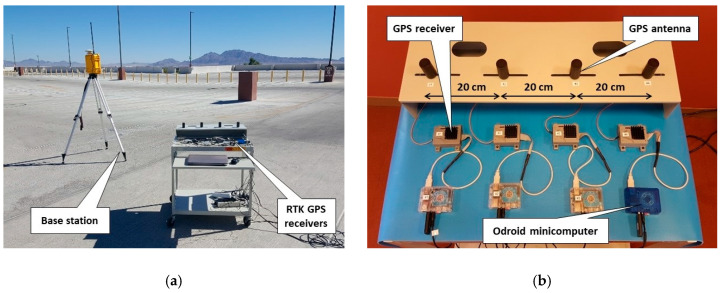
(**a**) Experiments using RTK GPS sensors; (**b**) antennas and receivers connected to UAS minicomputers.

To experimentally evaluate the performance of RTK GPS with multiple UASs, the fixed-position base station and four RTK “rovers” were set up, as shown in Figure 8a. The RTK GPS antennas of four rovers were equally spaced: 20 cm between the antennas, as displayed in Figure 8b. The Odroid minicomputers were running Linux (Ubuntu OS), ROS, and the application programing interface to access GPS data. The baseline position was related to coordinates of the base station.

Standard deviation σ (68% probability) of the position of four GPS units was measured as 2.5 mm, and 5.5 mm for the altitude. The RTK GPS positioning accuracy in a cluttered environment of the university campus was measured within 20 mm. The UAS position and altitude data measured using the RTK GPS technology with such accuracy enables high fidelity mapping of radiation data and allows for using it in cooperative sensing scenarios that involve multiple UASs.

### 3.2. Radiation Measurements

**Figure 9:**
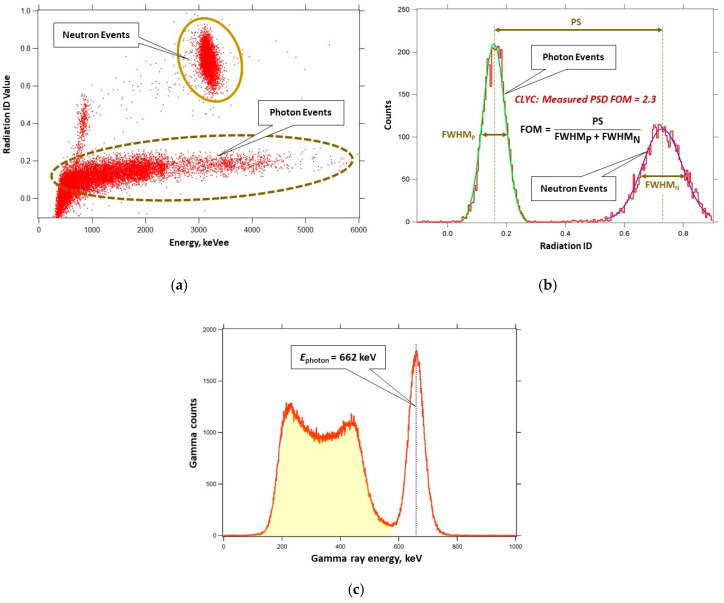
(**a**) Neutron/gamma pulse shape discrimination diagram of the CLYC sensor measurements in a mixed radiation flux using a PuBe source; (**b**) pulse shape discrimination (PSD) figure of merit measurement for CLYC sensor (figure of merit (FOM) = 2.3); (**c**) gamma spectrum recorded by the CLYC sensor for a ^137^Cs source (measured full widths at a half maximum (FWHM) energy resolution is <5% at 662 keV).

The CLYC sensor was tested for simultaneous measurements of gamma rays and neutrons. A plot of neutron/gamma PSD experiments using the CLYC sensor with a PuBe (α,*n*) source is presented in Figure 9a. The cloud of points indicative of neutron-induced reactions is centered around 3 MeVee (electron equivalents). This plot shows excellent separation of neutron and gamma events. A figure of merit (FOM) of neutron/gamma PSD [43] for CLYC was measured using a plot of the sensor’s counts versus the radiation IDs: the peak separation value was divided by the sum of full widths at a half maximum (FWHM) of neutron and gamma ray peaks, resulting in an FOM value of 2.3. Figure 9b illustrates the FOM measurement. The PSD algorithm was programmed using C language as a function in ROS to automatically process the measured waveforms.

The gamma-induced CLYC signals were separated from neutron signals using PSD analysis function and used to plot a gamma energy spectrum. The spectrum of gamma rays emitted by a ^137^Cs gamma source measured using the CLYC sensor is shown in Figure 9c.

The gamma ray FWHM energy resolution of the CLYC sensor was measured as less than 5% at 662 keV (see Table 1) using a ^137^Cs source. To determine the peak’s centroids and intensities for further identification of gamma sources, the spectrum should be automatically processed using spectral analysis algorithms [44,45,46]. Considering the limitations of the UAS’s Odroid minicomputer, a peak analysis algorithm based on the Mariscotti method [47,48] was programmed as a function in ROS.

Functions of PSD and spectral analysis programmed in ROS allow automatic neutron/gamma sorting of all measured signals, and identification and quantification of gamma emitters using the gamma signals only. These operations are performed onboard UASs, and the results of analysis (neutrons count rates, isotopes emitting gamma rays and their intensities) supplied with time of measurement and its GPS position are streamed to the ground station.

### 3.3. Radiation Damage Evaluation

**Figure 10:**
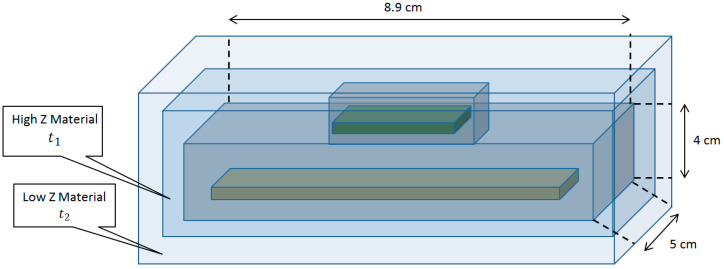
Shielding of the Pixhawk UAS controller.

The model of the UAS controller was designed in FLUKA. Shielding composed of layers of low-Z and high-Z materials was added around the electronic components to lower the DPA. A scheme of the shielding is shown in Figure 10. The following cases of the controller’s shielding were analyzed.

- Case 0: *t*_1_ = 0 mm, *t*_2_ = 0 mm. The DPA per incident gamma ray and DPA per incident neutron were calculated without shielding.
- Case 1: *t*_1_ = 0 mm, *t*_2_ = 5 mm. A single layer of polyethylene was used. The added weight of the polyethylene to the robotic platform would be 98 g.
- Case 2: *t*_1_ = 1 mm, *t*_2_ = 0 mm. A single layer of lead was used to shield the controller. The weight of the added lead would be 227 g.
- Case 3: *t*_1_ = 1 mm, *t*_2_ = 5 mm. Two layers—lead and polyethylene—were used. The added weight of the two-layer shielding would be 340 g.

The uniform beam was set as incident normally to the surface of the component from the bottom, irradiating the whole surface area of the component and the shielding. The statistical errors of the DPA Monte Carlo modeling using FLUKA were below 5% for 1 million simulated neutrons or photons in the beam. The DPA results are shown in Table 2 for neutrons, and in Table 3 for gamma rays, for the whole volume of the controller.

DPA increases with the energy of radiation. For the unshielded case, the DPA per incident photon increased 12.5 times when the energy increased from 1 MeV to 3 MeV. The DPA/neutron increased 1.4 times for neutron’s energy increase from 1 MeV to 3 MeV.

Case 3 showed the least values of DPA per an incident photon and neutron; however, this case added the most payload. When the shielding layers of case 3 were alternated (i.e., 1 mm of lead in the outer layer and 5 mm of polyethylene in the inner layer), the DPA due to neutron irradiation increased up to 20%, and the DPA due to gamma irradiation decreased by 12%. High-Z material in the inner layer attenuated secondaries generated by neutrons scattered by low-Z material in the outer layer.

**Figure 11:**
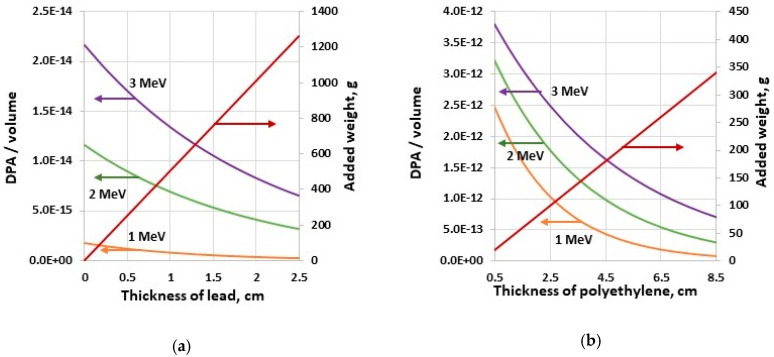
Parametric study of the shield in the bottom layer configuration for the incident (**a**) 10^8^ cm^−2^ 1 MeV photon flux and (**b**) 10^8^ cm^−2^ 1 MeV neutron flux.

Because the photon damage is three orders of magnitude less than the neutron damage for the same fluence, the use of heavy shielding material, such as lead, appears not practical for a mixed flux of neutrons and gamma rays of similar intensity due to a significant UAS payload increase. Thus, a single layer of a low-Z shield is a solution in this case. In remote sensing tasks, the UAS can be positioned above a radiation source, thus the radiation is incident primarily on the bottom of the robot. Placement of the shielding only on the bottom of electronic parts, not the sides and the top, allows decreasing of the added weight significantly. Parametric study of the bottom shield configuration for the controller (see Figure 11a,b) indicates that the DPA in the volume of the component decreases exponentially, while the shielding weight increase linearly. For 1 MeV gamma flux of 10^8^ cm^−2^ incident in the bottom of the controller, the 1-mm-thick lead shield decreases DPA/volume by only 7.5%, but the added weight is 50.5 g. The 5 mm lead layer decreases the DPA in the volume of the component by 32% with the added weight of 252 g. The 5 mm polyethylene layer decreases DPA/volume by 20% for 1 MeV neutron flux of 10^8^ cm^−2^, with added payload of 20 g. The 25 mm layer of polyethylene decreases DPA by 66%, adding the 100 g weight. The weight limit for shielding establishes the damage level for the UAS electronics. The energy required to displace an atom from its original lattice site is a threshold displacement energy [49,50]. DPA represents how many times an atom has been displaced from the lattice site, and this unit is directly related to the number of produced Frenkel pairs. Threshold energy values for various materials vary from 20 eV to 100 eV.

The threshold displacement energy for silicon in electronic components is 25 eV. The component will start failing once the accumulated damage level reaches about 10% of atoms displaced in the volume of the component.

**Figure 12:**
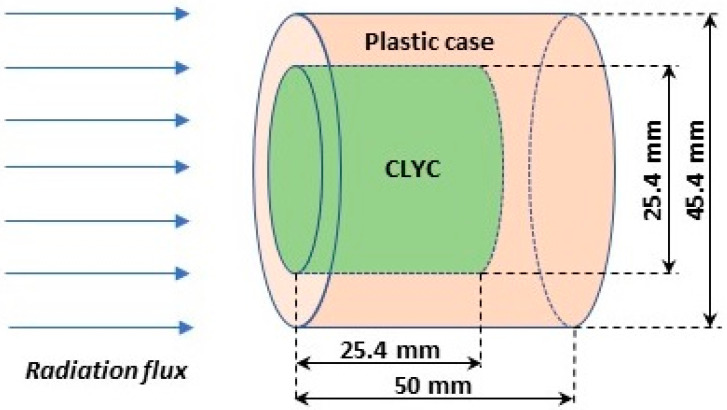
Schematics of the CLYC model in FLUKA.

When the ionizing radiation encounters the sensor’s material, a signal is generated. In UAS-based source searching and monitoring, the radiation sensor can be moved to areas where the contamination is unknown. Therefore, there is a risk of the radiation sensor’s material becoming damaged [51] by radiation, just like other components on the robotic platform. The CLYC sensor was modeled using FLUKA to evaluate the effect of the radiation damage by neutrons and gamma rays. Schematics of the CLYC model are shown in Figure 12. The CLYC material is shown in the green color, and a plastic casing surrounding the scintillator is shown in orange. The incident radiation beam is normal to the front face of the CLYC cylinder.

Photons and neutrons of energy 1 MeV, 2 MeV, and 3 MeV were modeled as incident normally into the face surface of the CLYC crystal. Radiation flux irradiated the surface uniformly. Table 4 and Table 5 show the results of the DPA calculations for the whole CLYC crystal for photons and neutrons, respectively. For each case, one million particles were utilized in simulations providing statistical errors below 5%. The gamma-induced damage of CLYC is two orders of magnitude less than for the silicon in electronic components, and the DPA/neutron is an order of magnitude lower. Also, CLYC is a scintillator with the optical readout, and can function if damaged. Therefore, the sensor material will better sustain the irradiation, unlike the electronics that must be protected.

Radiation- and energy-dependent DPA calculations allow estimation of the time of safe operation of the UAS under irradiation conditions of a specific mission and an applicable shielding design of vulnerable parts.

## 4. Conclusions

An ambient temperature neutron/gamma CLYC sensor was designed and integrated with the octocopter UAS platform. This sensor allows for simultaneous measurements of gamma rays and neutrons, with the effective separation of two radiations with the PSD figure of merit of 2.3. The developed sensor enables gamma spectroscopy with the energy resolution less than 5% for 662 keV photons. The CLYC sensor was integrated with the UAS as a “plug and fly” module, using Robot Operating System permitting a user-friendly deployment of the system in the field conditions. Functions for the onboard sensor’s waveform analysis, using PSD, gamma spectral analysis, and data fusion to add the high precision RTK GPS information and time stamps to the measured radiation data, were programmed in the ROS framework. The measured UAS positioning accuracy in the clustered environment using the RTK GPS system was 2 cm, enabling high fidelity spatial radiation mapping and cooperative sensing using multiple drones.

Radiation damage to UAS electronic parts and CLYC sensor material due to gamma and neutron irradiation was evaluated to assist in planning of the drone operations. The FLUKA code was utilized to analyze values of displacement per atom in components of the UAS. The radiation shielding options using layers of low-Z and high-Z materials were studied. The photon damage of electronic component was three orders of magnitude less than the neutron damage for the same fluence. Therefore, lead is not practical shielding for a mixed flux of neutrons and gamma rays of similar intensity, due to a substantial added weight. A single layer of a low-Z shield is appropriate in this case. In scenarios of UAS positioning above a source, radiation is incident primarily on the bottom of the drone. Protecting only the bottom of electronics allows decreasing of the payload significantly. The damage of CLYC crystal due to photon irradiation is two orders of magnitude less than the silicon damage in electronics, and the neutron-induced DPA is an order of magnitude lower. Hence, the scintillator crystal will tolerate the irradiation better, unlike the electronics that require shielding.

## Figures and Tables

**Table 1:** Gamma ray FWHM energy resolution of the CLYC sensor.

Photon Energy, MeV	FWHM Energy Resolution (%)
0.662	5.0
1.173	3.6
1.332	3.3

**Table 2:** Radiation damage due to neutron irradiation of the controller.

Neutron Energy, MeV	DPA per Incident Neutron (×10^−22^)			
	Case 0	Case 1	Case 2	Case 3
1.0	6.9	6.0	4.6	3.5
2.0	8.4	6.4	5.3	4.1
3.0	9.5	7.3	6.8	5.3

**Table 3:** Radiation damage due to gamma ray irradiation of the controller.

Photon Energy, MeV	DPA per Incident Photon (×10^−25^)			
	Case 0	Case 1	Case 2	Case 3
1.0	3.9	3.0	0.28	0.09
2.0	26.0	14.6	0.47	0.27
3.0	48.6	38.9	2.9	0.35

**Table 4:** Radiation damage due to photon irradiation of the CLYC crystal.

Photon Energy, MeV	DPA per Incident Photon (×10^−27^)
1.0	1.8
2.0	8.2
3.0	14.4

**Table 5:** Radiation damage due to neutron irradiation of the CLYC crystal.

Neutron Energy, MeV	DPA per Incident Photon (×10^−23^)
1.0	5.3
2.0	8.0
3.0	10.3
